# Expression of Cell-Surface Marker ABCB5 Causes Characteristic Modifications of Glucose, Amino Acid and Phospholipid Metabolism in the G3361 Melanoma-Initiating Cell Line

**DOI:** 10.1371/journal.pone.0161803

**Published:** 2016-08-25

**Authors:** Norbert W. Lutz, Pallavi Banerjee, Brian J. Wilson, Jie Ma, Patrick J. Cozzone, Markus H. Frank

**Affiliations:** 1 Centre de Résonance Magnétique Biologique et Médicale, Unité Mixte de Recherche 7339 Centre National de la Recherche Scientifique, Faculté de Médecine de la Timone, Aix-Marseille Université, Marseille, France; 2 Transplant Research Program, Boston Children's Hospital, Harvard Medical School, Boston, Massachusetts, United States of America; 3 Harvard Skin Disease Research Center, Department of Dermatology, Brigham and Women’s Hospital, Harvard Medical School, Boston, Massachusetts, United States of America; 4 Singapore Imaging Consortium, Agency for Science, Technology and Research, Singapore; 5 School of Medical Sciences, Edith Cowan University, Joondalup, Western Australia, Australia; East Tennessee State University, UNITED STATES

## Abstract

We present a pilot study aimed at determining the effects of expression of ATP-binding cassette member B5 (ABCB5), a previously described marker for melanoma-initiating cells, on cellular metabolism. Metabolic profiles for two groups of human G3361 melanoma cells were compared, i.e. wildtype melanoma cells with intact ABCB5 expression (ABCB5-WT) and corresponding melanoma cell variants with inhibited ABCB5 expression, through shRNA-mediated gene knockdown (ABCB5-KD). A comprehensive metabolomic analysis was performed by using proton and phosphorus NMR spectroscopy of cell extracts to examine water-soluble metabolites and lipids. Parametric and non-parametric statistical analysis of absolute and relative metabolite levels yielded significant differences for compounds involved in glucose, amino acid and phospholipid (PL) metabolism. By contrast, energy metabolism was virtually unaffected by ABCB5 expression. The sum of water-soluble metabolites per total protein was 17% higher in ABCB5-WT vs. ABCB5-KD G3361 variants, but no difference was found for the sum of PLs. Enhanced abundance was particularly pronounced for lactate (+ 23%) and alanine (+ 26%), suggesting an increase in glycolysis and potentially glutaminolysis. Increases in PL degradation products, glycerophosphocholine and glycerophosphoethanolamine (+ 85 and 123%, respectively), and redistributions within the PL pool suggested enhanced membrane PL turnover as a consequence of ABCB5 expression. The possibility of glycolysis modulation by an ABCB5-dependent IL1β-mediated mechanism was supported by functional studies employing monoclonal antibody (mAb)-dependent ABCB5 protein inhibition in wildtype G3361 melanoma cells. Our metabolomic results suggest that the underlying biochemical pathways may offer targets for melanoma therapy, potentially in combination with other treatment forms.

## Introduction

Tumor-initiating cells, also known as cancer stem cells (CSC), have been identified in several human malignancies, including melanoma. We recently found that ABCB5 (ATP-Binding Cassette, Sub-Family B (MDR/TAP), Member 5), a cell-surface marker for melanoma-initiating cells (MICs) [1], is functionally required for MIC-driven melanoma growth [2]. CSC typically reside in hypoxic niches of tumors, where they are often protected against anticancer treatment effects [3]. Hypoxia-inducible factors (HIFs) regulate cellular biochemistry, notably glycolysis, energy and membrane phospholipid (PL) metabolism [4–6]. This prompted us to investigate, by ^1^H and ^31^P NMR spectroscopy of cell extracts, whether these and other metabolic pathways are directly altered by ABCB5 expression, i.e. if the functional role of ABCB5 may include 'priming' specific metabolic processes essential to melanoma cell survival and proliferation. Such processes may become important as potential targets for more efficient anticancer therapy.

ABCB5 is a subfamily B multidrug resistance (MDR) member of the ABC superfamily of active transporters. A limited number of previously published studies on MDR and the expression of ABC transporters other than ABCB5 have reported the presence of significant effects on cancer cell metabolism. However, these effects varied as a function of the ABC transporter and cell line in question. Nonetheless, most stem-like cancer cells appear to have several metabolic traits in common, the most prevalent being increased glycolysis [7]. The latter effect has been demonstrated for glioma stem cells and for side populations with elevated ABCG2 expression of a variety of human cancer cell lines: non-small cell lung cancer (NSCLC) A549, lung cancer H460 and colon cancer LoVo [8]. Increased glycolysis has also been observed for breast cancer stem cells obtained from patients [9], for the CD133-positive colon carcinoma cell line Colo205 [10], for a murine model of LDH (lactate dehydrogenase)-A-expressing NSCLC [11], and for glioma stem-like cells [12], although there is a report of glioma stem cells with reduced glycolysis and increased oxidative phosphorylation [13]. Glycolysis is closely linked to energy metabolism. We have previously demonstrated, in a comparison between two renal cell carcinoma (RCC) cell lines, that the high-MDR1 cell line, KTCTL-26, had a higher energetic status and an increased level of the high-energy metabolite, phosphocreatine (PCr), when compared with the low-MDR1 cell line, KTCTL-2 [14]. This was in agreement with previously reported ^31^P NMR studies of various MDR1-transfected cancer cells [15]. By contrast, ATP, another high-energy metabolite, was lower in KTCTL-26 than in KTCTL-2, which was linked to KTCTL-2 drug resistance effects other than MDR [14]. Similarly, levels of phosphodiesters (PDE), which are known to be phospholipid degradation products, were decreased in KTCTL-26 *vs*. KTCTL-2 cells, although low PDE are generally associated with low MDR1 expression [15–17]. Again, this effect was ascribed to KTCTL-2 drug resistance effects other than MDR [14]. In summary, work previously carried out by us and others suggests that the presence of multiple, redundant mechanisms of resistance may be at the origin of confounding parameters when metabolic effects of ABC transporter-expressing cells are studied. Consequently, definite results for specific MDR transporters can only be gained under highly controlled conditions. For this reason, we present here first unambiguous metabolic results for a well-established human melanoma model, the G3361 cell line [1], with either intact ABCB5 expression in its ABCB5 wildtype state, or inhibited ABCB5 expression in its shRNA-mediated gene knockdown state, established according to methodologies described previously [2].

## Materials and Methods

### Cell culture and extraction

ABCB5-KD and ABCB5-WT G3361 melanoma cells with inhibited and intact ABCB5 expression, respectively, were generated as described previously as stable shRNA pSUPER-retro-puro-ABCB5-KD cell populations or the respective shRNA-control cell populations, using previously validated ABCB5 shRNA targeting or control shRNA sequences [2]. Knockdown levels and other functional details of these cells have been reported previously [1,2]. ABCB5-WT and ABCB5-KD G3361 melanoma cells (n = 4 each) were analyzed for differences in metabolite profiles, with an emphasis on energy and PL metabolism. For each analysis, approximately 3×10^7^ cells were cultured to near confluency in flasks containing RPMI 1640 medium (Lonza, Basel, Switzerland) supplemented with 10% fetal bovine serum (Gibco Life Technologies, Carlsbad, USA) under standard conditions (37°C, 5% CO_2_ atmosphere). Cells were quickly rinsed with ice-cold saline and frozen by pouring liquid nitrogen into each flask. Upon nitrogen evaporation, 4 mL ice-cold methanol was added, which froze rapidly to the flask bottom. Cells were scratched into the methanol as it began to thaw. The melted mixture was transferred to a glass tube and incubated on ice for 15 min. Then, 4 ml of ice-cold CHCl_3_ and 4 ml of ice-cold water were added sequentially, with thorough vortexing after each addition. Samples were placed at -20°C overnight for phase separation, which was completed by subsequent centrifugation by analogy to a procedure described previously [18]. The protein precipitate was kept at -80°C for total-protein determination.

### Sample preparation for metabolomic NMR spectroscopy

Solvents were removed from the chloroform/methanol and water/methanol phases as described elsewhere [18], and the dried samples were stored at -80°C. For NMR spectroscopy, lipids were dissolved in an appropriate amount (700 to 1400 μ l) of a ^2^H-chloroform/methanol/ water solution as described elsewhere [19, 20], to yield solutions of similar phospholipid concentrations for all samples. Water-soluble metabolites were redissolved in 700 μ l D_2_O, and pH was adjusted to ca. 7.0. The sample was placed in a 5-mm Wilmad NMR tube (528-PP; Carlo Erba-SDS, Val de Reuil, France) containing a stem coaxial insert (2 mm O.D.) filled with a D_2_O solution (pH 7.7) of 2 mM sodium 3-(trimethylsilyl)-2,2',3,3'-tetradeuteropropionate (TSP-d_4_) for ^1^H NMR, or with aqueous 20 mM methylenediphosphonate (MDP) for ^31^P NMR, for chemical-shift referencing and quantitation. Chemicals were purchased from Sigma-Aldrich (Saint Quentin, Fallavier, France), except for a number of phospholipids used for ^31^P NMR signal assignment (Doosan Serdary Research, Toronto, ON, CA).

### NMR spectroscopy

^1^H and ^31^P NMR spectra at 400.1 and 162.0 MHz, respectively, were obtained on a 9.4 T AVANCE 400 WB FT-NMR spectrometer from Bruker (Wissembourg, France), and analyzed with Bruker’s Topspin software (further technical details have been described elsewhere [21]). NMR signals were assigned based on previous work [21] and on spiking of extracts with original compounds where necessary. Absolute metabolite concentrations are given as nmol/mg total protein. In addition, relative molar concentrations (rel. conc.) were calculated by dividing, for each spectrum, the integral of a characteristic metabolite NMR resonance, m, by the total integral of all metabolites from the same spectrum, as in Eq 1:

$$\text{rel.conc.(m)}=\frac{{\text{int}}_{\text{metabolite,m}}}{\sum_{\text{i}=1}^{\text{n}}{\text{int}}_{\text{metabolite,i}}}$$ (1)

Here, int_metabolite,m_ is the integral of the characteristic resonance of metabolite m; int_metabolite,i_ represents the sum of all integrals of metabolite i; and n is the total number of metabolites producing measurable spectral lines.

Comparisons of relative metabolite concentrations between groups have the advantage of directly revealing redistributions within the tissue metabolite pools, irrespective of differences between pool sizes. In addition, evaluating relative concentrations eliminates potential errors introduced by sample handling such as weighing or pipetting, as any loss in biological material that might occur would affect all metabolites of a given sample to the same extent, and would, therefore, not change relative concentrations. Obviously, the latter advantage may even be relevant in those cases where the total metabolite pool does not vary significantly between groups. However, it is evident that determinations of absolute concentrations are indispensable for evaluating absolute differences in metabolite levels between groups.

### Quantitation of total protein

For each extracted sample, total protein was determined in triplicate using a bicinchoninic acid (BCA) protein assay kit (Pierce Biotechnology, Rockford, lL, USA). Absorbance at 562 nm was determined using a Uvikon 930 spectrophotometer (Kontron Instruments, Watford, UK).

### Statistical evaluation

The statistical methods used to detect significant differences between groups included parametric and nonparametric tests (Prism 5.0, GraphPad, La Jolla, CA, USA). Parametric tests such as Student's t test and analysis of variance with parametric post-hoc analysis are appropriate where the conditions of equal variances and normal distribution are met. However, tests for normal distribution do not give meaningful results when applied to small sample groups [22, 23], as is the case in this study. To minimize any bias due to a lack of statistical power, we therefore tested the outcome of both parametric and non-parametric methods. In virtually all cases, these methods yielded consistent results for statistical significance of differences in metabolite concentrations. Only a few inter-group comparisons for select low-abundance metabolites yielded statistical significance based on parametric tests while non-parametric tests exhibited borderline significance (0.05 < p < 0.1), due to the fact that non-parametric tests such as the Mann-Whitney *U* or Kruskal-Wallis test (that are intrinsically conservative) were not sufficiently powerful to unambiguously demonstrate significant differences.

### Western Blot

Whole cell lysates from human G3361 melanoma cells were run in duplicate on SDS polyacrylamide gel and transferred to a polyvinylidene difluoride membrane (GE Healthcare). The membranes were incubated with primary antibodies, i.e. anti-Hexokinase I (clone C35C4), anti-Hexokinase II (clone C64G5), anti-PKM1/2 (clone C103A3), anti-PFKP (clone D4B2), anti-Pyruvate Dehydrogenase (Clone C54G1), anti-GAPDH (clone D16H11), anti-HIF-1α (clone D2U3T), or anti-β-Actin (clone 8H10D10) (Cell Signaling Technology), and subsequently incubated with peroxidase-linked secondary antibody. The reactive bands were detected by using chemiluminescent substrate (Thermo Scientific). Quantitative Western Blot signal intensity analysis was performed using Image StudioTM Lite Software (LI-COR Biosciences).

## Results

### General metabolic trends

Based on metabolite profiles acquired for extracts of ABCB5-WT and ABCB5-KD G3361 melanoma cells, 45 metabolite concentrations were obtained for each sample group (see Figures A-C in S1 File for representative metabolite spectra for extracts of ABCB5-expressing G3361 cells; Tables A-D in S1 File). Five of these concentrations relate to PL subclasses consisting of at least two separately quantifiable PL subgroups each. In addition, 13 partially assigned subclasses of low-concentration PLs, whose exact identification is currently underway, were detected and quantified. This resulted in 67 nominal concentration values and a total of 157 absolute and relative metabolic parameters. Additional metabolites were occasionally detected in select cell extracts, *e*.*g*., branched organic acids such as β-hydroxyisobutyrate. However, we only included those compounds in our quantitative analysis that were above the detection threshold for most samples.

**Fig 1:**
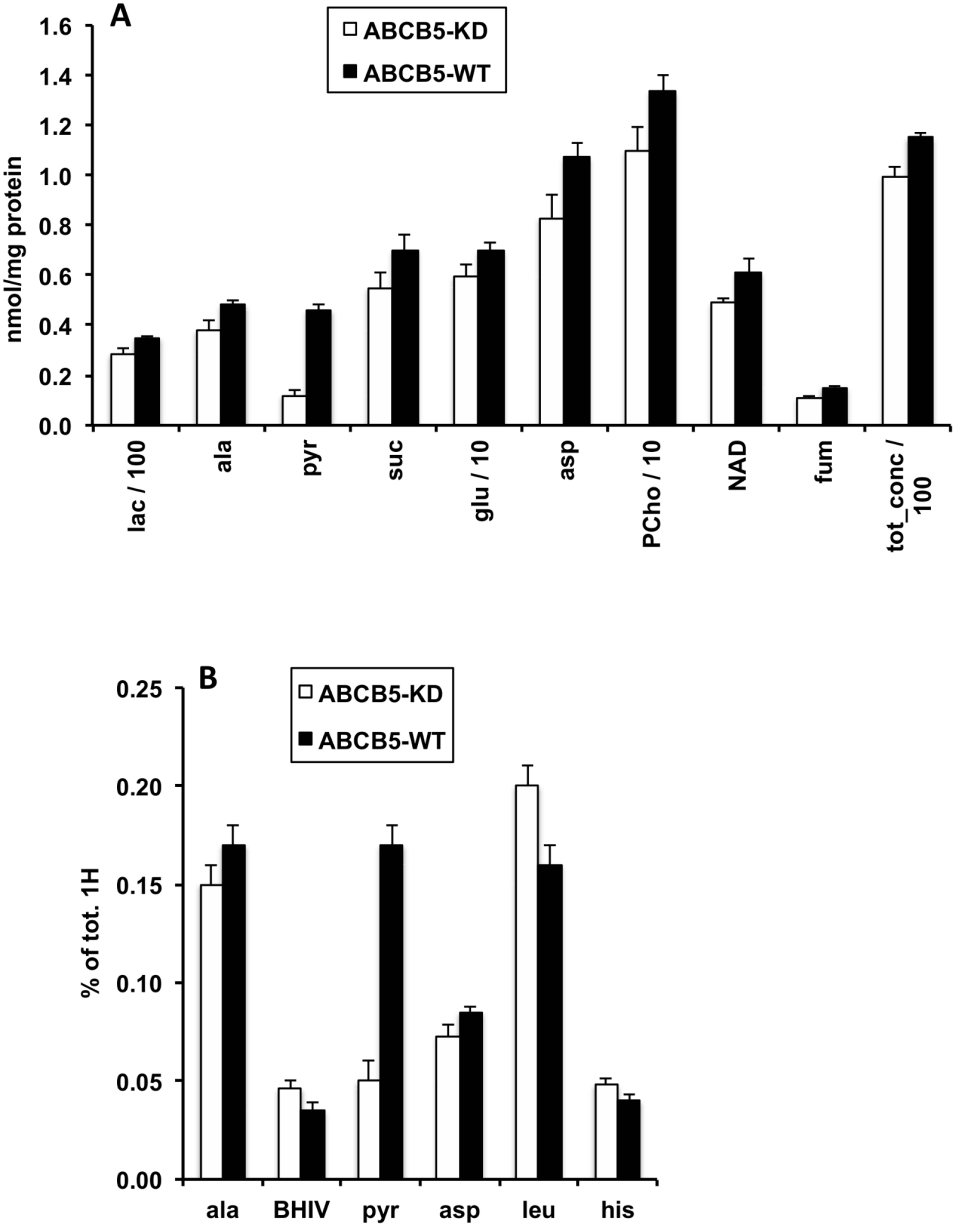
Effects of ABCB5 expression on levels of water-soluble metabolites. Values for human G3361 melanoma cells, measured by ^1^H NMR spectroscopy of cell extracts (aqueous phase), are given as means ± SEM, with n = 4 for each group. Open bars, ABCB5-KD cells; full bars, ABCB5-WT cells. Differences between ABCB5-KD and ABCB5-WT cells were significant; detailed statistical results for these metabolites are provided in Table 1. A: Absolute metabolite concentrations, nmol/mg protein. B: Relative metabolite concentrations, % of total ^1^H NMR metabolite signals. Abbreviations: lac, lactate; ala, alanine; pyr, pyruvate; suc, succinate; glu, glutamate; asp, aspartate; PCho, phosphorylcholine; NAD, nicotinamide adenine dinucleotide; fum, fumarate; BHIV, β-hydroxy isovalerate; leu; leucine; his, histidine; tot_conc, total concentration of all water-soluble metabolites measured by ^1^H NMR spectroscopy.

**Fig 2:**
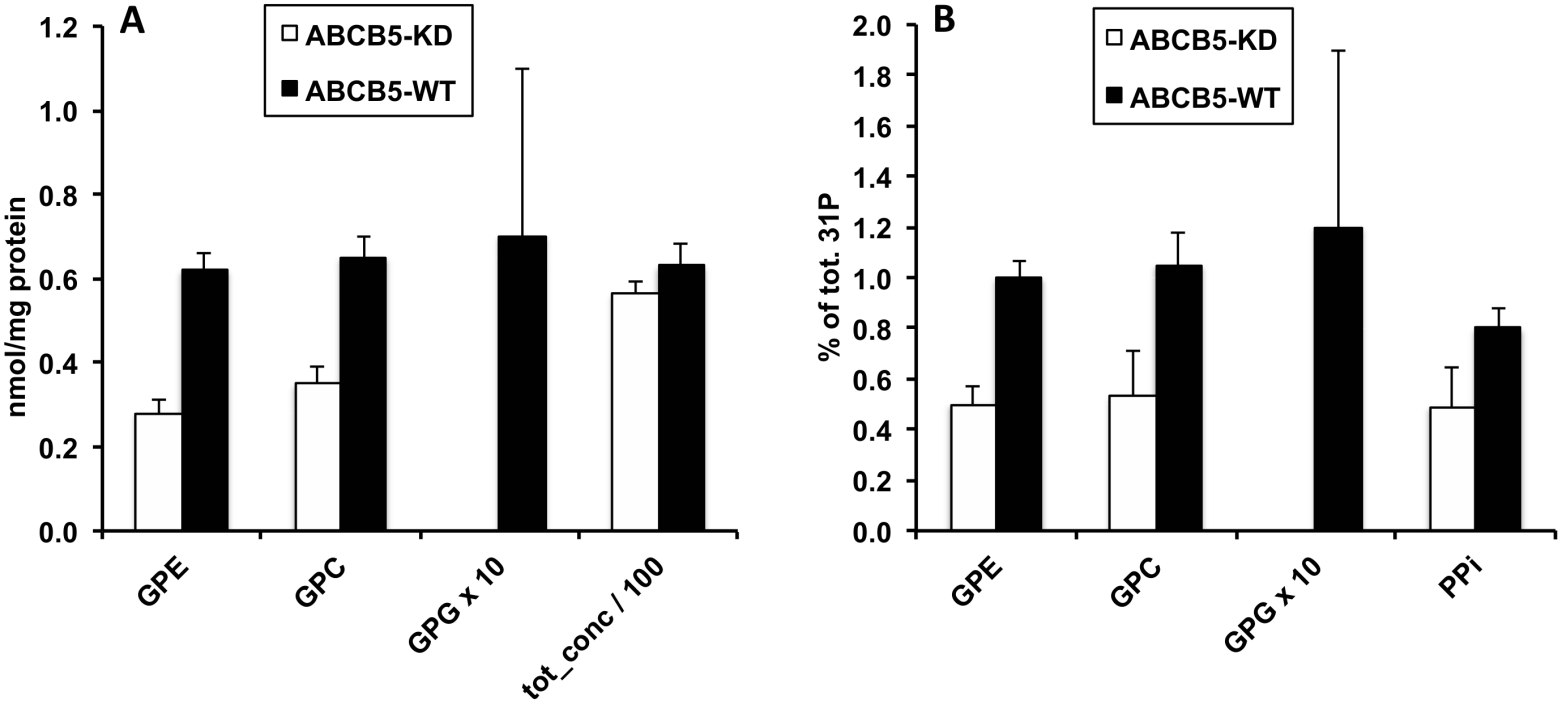
Effects of ABCB5 expression on levels of phosphorylated water-soluble metabolites. Values for human G3361 melanoma cells, measured by ^31^P NMR spectroscopy of cell extracts (aqueous phase), are given as means ± SEM, with n = 4 for each group. Open bars, ABCB5-KD cells; full bars, ABCB5-WT cells. Differences between ABCB5-KD and ABCB5-WT cells were significant; detailed statistical results for these metabolites are provided in Table 2. A: Absolute metabolite concentrations, nmol/mg protein. B: Relative metabolite concentrations, % of total ^31^P NMR metabolite signals Abbreviations: GPE, glycerophosphorylethanolamine; GPC, glycerophosphorylcholine; GPG, glycerophosphorylglycerol; PPi, pyrophosphate; tot_conc, total concentration of all water-soluble metabolites measured by ^31^P NMR spectroscopy.

**Fig 3:**
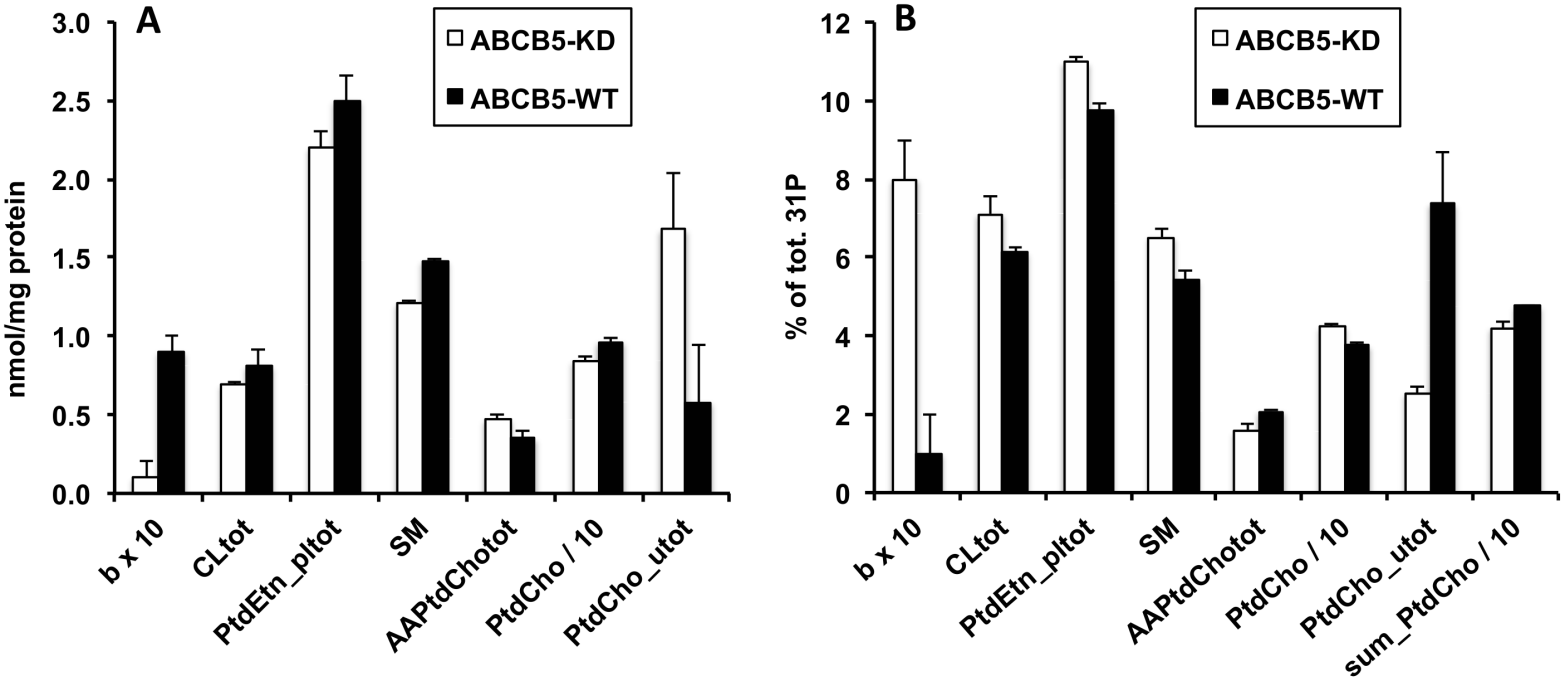
Effects of ABCB5 expression on phospholipid levels. Values for human G3361 melanoma cells, measured by ^31^P NMR spectroscopy of cell extracts (organic phase), are given as means ± SEM, with n = 4 for each group. Open bars, ABCB5-KD cells; full bars, ABCB5-WT cells. Differences between ABCB5-KD and ABCB5-WT cells were significant; detailed statistical results for these phospholipids are provided in Table 3. A: Absolute phospholipid concentrations, nmol/mg protein. B: Relative phospholipid concentrations, % of total ^31^P NMR phospholipid signals. Abbreviations: b, unassigned phospholipid, most likely a cardiolipin-derived phospholipid subgroup; CL_tot_, total cardiolipin; PtdEtn_pl_tot_, total ethanolamine plasmalogen; SM, sphingomyelin; AAPtdCho_tot_, total alkyl-acyl-phosphatidylcholine; PtdCho, phosphatidylcholine; PtdCho_u_tot_, total phosphatidylcholine u derivatives; sum_PtdCho, sum of PtdCho and all PtdCho derivatives. See EXCEL file S1 Table for further explanations.

ABCB5-WT G3361 melanoma cells were characterized by a higher total amount of water-soluble metabolites per total protein than ABCB5-KD G3361 cells (Fig 1, Table 1). This difference was statistically significant for the sum of metabolites, totH, detected by their proton (^1^H) NMR signals (+ 20%), whereas only a trend was found for the sum of phosphorylated metabolites, totP, detected by their phosphorus (^31^P) NMR signals (+ 14%, Fig 2 and Table 2). No difference was found for the sum of PLs per total protein (Fig 3). Characteristic differences in individual metabolite concentrations due to ABCB5 expression will be described in the following paragraphs. Remarkably, energy metabolites (ATP, ADP, phosphocreatine) and many organic acids, including most amino acids analyzed, did not exhibit significant changes upon ABCB5 expression, as detailed below.

**Table 1:** Significant differences between levels of water-soluble metabolites in ABCB5-WT and ABCB5-KD G3361 melanoma cells based on ^1^H NMR spectroscopy of cell extracts.

Absolute concentrations, nmol/mg total protein					
metabolite	lac*	ala	pyr**	suc	glu
mean ± SEM					
ABCB5-WT	34.97 ± 0.81	0.48±0.02	0.46±0.02	0.70±0.06	6.95 ± 0.32
ABCB5-KD	28.45 ± 2.18	0.38±0.04	0.12±0.02	0.55±0.06	5.94 ± 0.45
p values					
Mann-Whitney	0.0571	0.1143	0.1000	0.2000	0.1174
t test	**0.0312**	0.0527	**0.0002**	0.0987	0.1143
metabolite	asp	PCho	NAD	fum	tot_conc
mean ± SEM					
ABCB5-WT	1.07±0.06	13.33 ± 0.69	0.61±0.06	0.15±0.01	115.0 ± 2.22
ABCB5-KD	0.83±0.09	11.00 ± 0.91	0.49±0.02	0.11±0.01	99.6 ± 4.12
p values					
Mann-Whitney	0.1134	0.2000	0.2000	0.0571	**0.0286**
t test	0.0731	0.0874	0.1137	**0.0448**	**0.0167**
Relative concentrations, % of total metabolite protons					
metabolite	ala	BHIV	pyr**		
mean ± SEM					
ABCB5-WT	0.17±0.01	0.035±0.004	0.17±0.01		
ABCB5-KD	0.15±0.01	0.046±0.004	0.05±0.01		
p values					
Mann-Whitney	0.2000	0.1143	0.1000		
t test	0.0729	0.1034	**0.0006**		
metabolite	asp	leu	his		
mean ± SEM					
ABCB5-WT	0.085±0.003	0.16±0.01	0.040±0.003		
ABCB5-KD	0.073±0.006	0.20±0.01	0.048±0.003		
p values					
Mann-Whitney	0.1143	**0.0286**	0.1143		
t test	0.1069	**0.0147**	0.1043		

Statistical significance was determined by Mann-Whitney and t tests (see Materials and Methods). For abbreviations see legend to Fig 1. Bold p values: significant (p<0.05); underlined p values: borderline significant (0.05<p<0.12).

*Includes minor contribution from threonine.

**Two outliers excluded; differences not significant when outliers included. Further metabolite levels based on ^1^H NMR are provided by Table A in S1 File.

**Table 2:** Significant differences between levels of phosphorylated water-soluble metabolites in ABCB5-WT and ABCB5-KD G3361 melanoma cells based on ^31^P NMR spectroscopy of cell extracts.

Absolute concentrations, nmol/mg total protein				
metabolite	GPE	GPC	GPG*	tot_conc
mean ± SEM				
ABCB5-WT	0.62±0.04	0.65±0.05	0.07±0.04	63.03±5.13
ABCB5-KD	0.28±0.03	0.35±0.04	n.d.	56.32±2.75
p values				
Mann-Whitney	**0.0286**	**0.0286**	n.d.	0.8857
t test	**0.0002**	**0.0049**	n.d.	0.2926
Relative concentrations, % of total metabolite phosphate				
metabolite	GPE	GPC	GPG*	PPi
mean ± SEM				
ABCB5-WT	1.00±0.07	1.05±0.13	0.12±0.07	0.80±0.08
ABCB5-KD	0.50±0.07	0.53±0.18	n.d.	0.49±0.16
p values				
Mann-Whitney	**0.0286**	**0.0286**	n.d.	0.1143
t test	**0.0019**	0.0607	n.d.	0.1311

Statistical significance was determined by Mann-Whitney and t tests (see Materials and Methods). For abbreviations see legend to Fig 2. Bold p values: significant (p<0.05); underlined p values: borderline significant (0.05<p<0.12).

*p values not determined as GPG levels were undetectably low in ABCB5-KD cells. Further levels of water-soluble metabolites based on ^31^P NMR are provided by Table B in S1 File.

### Effects of ABCB5 expression on water-soluble metabolites

The higher levels of absolute metabolite concentrations observed for ABCB5-WT *vs*. ABCB5-KD cells were particularly noticeable for the glycolytic metabolites, lactate (+ 23%) and pyruvate (+ 286%), as well as for the Krebs cycle intermediate, fumarate (+ 38%), for the amino acid, alanine (+ 26%), and for the PL catabolites, glycerophosphoethanolamine (GPE, + 123%) and glycerophosphocholine (GPC, + 85%). All these differences were statistically significant (p < 0.05, t-test; Tables 1 and 2). Further increases (by 17 to 29%) were close to statistical significance (0.05 < p < 0.12); these concern the amino acids, glutamate and aspartate, the Krebs cycle intermediate, succinate, the PL metabolite, phosphocholine (PCho), and the coenzyme, nicotinamide adenine dinucleotide (NAD). Another PL catabolite, glycerophosphoglycerol (GPG), was detectable and quantifiable in the ABCB5-WT group, but not in the ABCB5-KD group. Actual PL concentrations are presented below (Table 3).

**Table 3:** Significant differences between phospholipid levels in ABCB5-WT and ABCB5-KD G3361 melanoma cells.

Absolute concentrations, nmol/mg total protein				
PL class	b	CL_tot_	PtdEtn_pl_tot_	SM
mean ± SEM				
ABCB5^+^	*0*.*01 ± 0*.*01*	0.69 ± 0.02	2.20 ± 0.11	1.21 ± 0.01
ABCB5^-^	0.09 ± 0.01	0.81 ± 0.10	2.50 ± 0.16	1.48 ± 0.03
p values				
Mann-Whitney	**0.0436**	0.4000	0.2286	0.0571
t test	**0.0053**	0.1969	0.1626	**0.0002**
metabolite	AAPtdCho_tot_	PtdCho	PtdCho_u_tot_	
mean ± SEM				
ABCB5^+^	0.47 ± 0.03	8.43 ± 0.27	1.68 ± 0.36	
ABCB5^-^	0.35 ± 0.05	9.65 ± 0.56	0.58 ± 0.04	
p values				
Mann-Whitney	0.2286	0.2286	0.0571	
t test	0.0784	0.0833	**0.0481**	
Relative concentrations, % of total PL phosphorus				
PL class	b	CL_tot_	PtdEtn_pl_tot_	SM
mean ± SEM				
ABCB5^+^	*0*.*10 ± 0*.*10*	6.14 ± 0.12	9.77 ± 0.15	5.41 ± 0.24
ABCB5^-^	0.80 ± 0.10	7.11 ± 0.45	10.98 ± 0.08	6.52 ± 0.31
p values				
Mann-Whitney	**0.0436**	0.0571	**0.0471**	0.0571
t test	**0.0046**	0.0610	**0.0014**	**0.0336**
PL class	AAPtdCho_tot_	PtdCho	PtdCho_u_tot_	sum_PtdCho
mean ± SEM				
ABCB5^+^	2.08 ± 0.06	37.51 ± 0.98	7.37 ± 1.32	47.57 ± 0.36
ABCB5^-^	1.55 ± 0.18	42.34 ± 0.81	2.54 ± 0.05	42.05 ± 1.27
p values				
Mann-Whitney	0.0571	0.0571	0.0571	0.0571
t test	**0.0213**	**0.0156**	**0.0273**	**0.0048**

Statistical significance was determined by Mann-Whitney and t tests (see Materials and Methods). Bold p values: significant (p<0.05); underlined p values: borderline significant (0.05<p<0.12). Sample sizes: n = 4 for ABCB5-WT; n = 3 for ABCB5-KD. Values in italics: PL class or subclass only detected in one extract of this group. This table also includes PL subclasses. For abbreviations see legend to Fig 1. Further phospholipid levels are provided by Tables C and D in S1 File.

All these variations reflect, to different degrees, the general increase in water-soluble metabolites per total protein, *i*.*e*., totH and totP, with ABCB5 expression. In addition, we were also interested in identifying directly those compound levels that exhibited significantly higher (or lower) inter-group differences than the global parameters, totH and totP. This information was obtained by studying relative metabolite levels, *i*.*e*. the percentages of ^1^H and ^31^P metabolite signals of total ^1^H and ^31^P signals, respectively. Relative pyruvate, GPC and GPE levels were significantly higher in ABCB5-WT *vs*. ABCB5-KD melanoma cells by a factor of three, two and two (respectively), whereas relative alanine and aspartate increases by about 15% were close to significance (0.05 < p ≤ 0.12; % of totH and totP, Tables 1 and 2). By contrast, relative levels of the amino acid, leucine, were significantly lower by about 20% in ABCB5-WT *vs*. ABCB5-KD cells, accompanied by less significant decreases for the branched-chain fatty acid, β-hydroxyisovalerate (BHIV), which is a leucine metabolite, and the aromatic amino acid, histidine (0.05 < p ≤ 0.12). Note that based on absolute concentrations, none of the latter three metabolites varied between groups. Taken together, these data demonstrate that ABCB5 expression, while causing a general increase in water-soluble metabolites, also generated a redistribution of the metabolite pool such that an accumulation of PL degradation products and some amino acids is favored over other organic acids. The final product of glycolysis proper is pyruvate, which is then either fed into the citric acid cycle for oxidation to CO_2_, or reduced to lactate or alanine. The fate of pyruvate is significantly influenced by the cellular oxygen status. Minor variations in cell handling during extract preparation may easily affect the oxygen status and, therefore, glucose metabolism. For better consistency, we excluded the pyruvate level outlier found in each group of ABCB5-WT and ABCB5-KD cells (Table 1), and the glucose level outlier found for the ABCB5-WT group (Table A in S1 File). Note that the pyruvate difference between ABCB5-WT and ABCB5-KD cells would not be significant without the omission of outliers.

In contrast to these variations, the concentrations of numerous important metabolites proved to be remarkably invariant to ABCB5 expression (Tables A and B in S1 File). In particular, the intracellular organic osmolytes, taurine and *myo*-inositol (myo-Ins), were unaffected by ABCB5 expression, with respect to both absolute and relative concentrations; both compounds are also known to be antioxidants. While myo-Ins is a glucose metabolite, the concentrations of glucose itself, and of another group of glucose metabolites (UDP-hexoses, predominantly consisting of UDP *N*-acetyl sugars) were also unchanged as a function of ABCB5 expression. The latter play an important role in intracellular signaling and nutrient-sensing, as UDP *N*-acetyl hexosamine synthesis integrates glucose, amino acid and lipid metabolism with nucleotide and energy metabolism. Furthermore, aliphatic and aromatic amino acids were mostly unaffected by ABCB5 expression; this applies to valine, isoleucine, tyrosine and phenylalanine, but also to the metabolites of the phosphagen system, creatine, phosphocreatine (PCr) and ATP. Taken together, our findings hint at a high degree of stability against perturbation by ABCB5 expression for osmoregulation, glucose and amino acid metabolism, in contrast with the results obtained for phospholipid metabolism.

### Functional blockade of ABCB5 inhibits key molecules of the glycolysis pathway through regulation of IL-1β signaling

**Fig 4:**
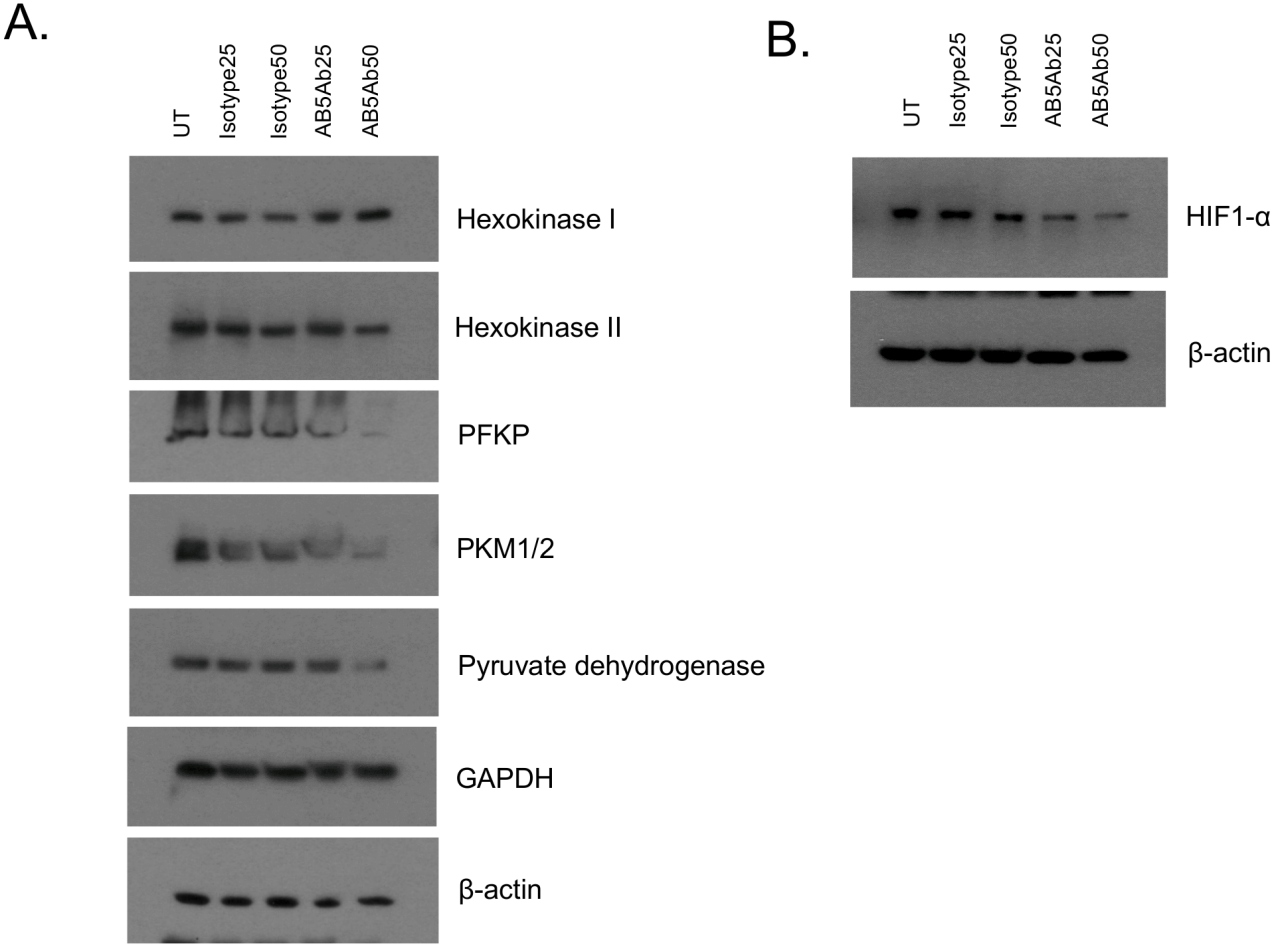
ABCB5 protein blockade inhibits key regulators of the glycolysis pathway. Human wildtype G3361 melanoma cells were treated with different concentrations of blocking anti-ABCB5 mAb clone 3C2-1D12 or corresponding doses of isotype-matched control mAb, or were left untreated for 48 h. Expression of Hexokinase 1, Hexokinase II, PKM1/2, PFKP, Pyruvate Dehydrogenase, GAPDH (A), HIF-1α (B) and β-Actin was analyzed by Western blot. Data shown are representative of three independent experiments.

**Fig 5:**
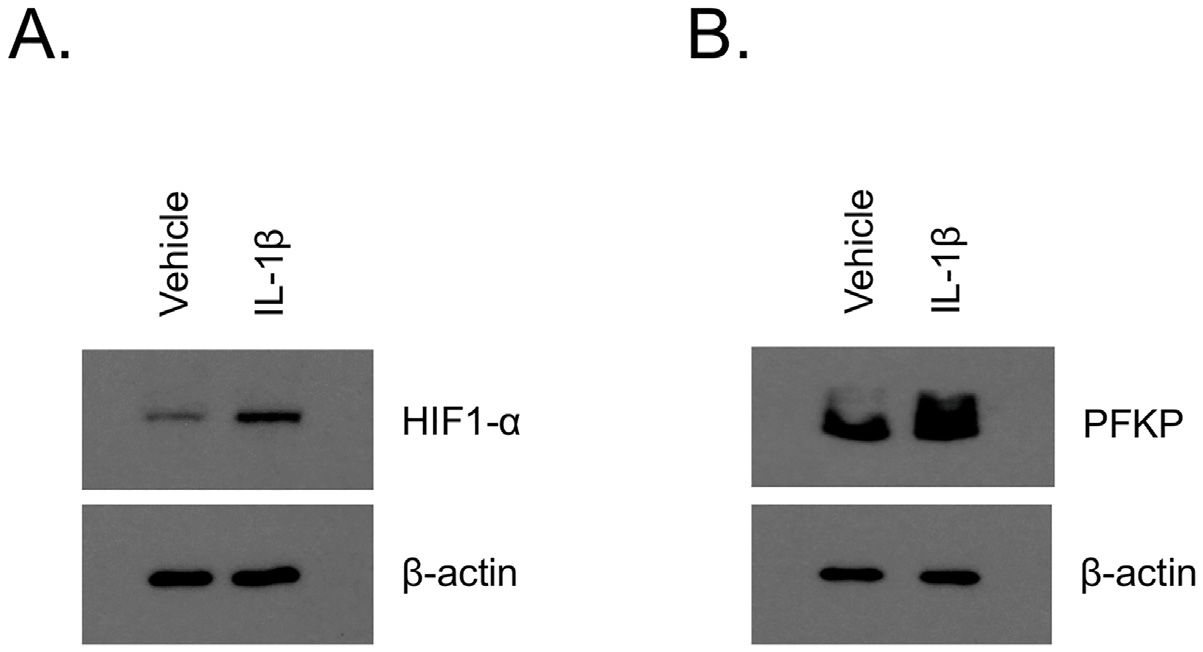
Treatment with exogenous IL-1β induces regulators of the glycolysis pathway. Human wildtype G3361 melanoma cells were treated with 10 ng/ml recombinant IL-1β or vehicle control. Lysates prepared from the cells were analyzed for the expression of HIF-1α (A), PFKP (B) and β-Actin by Western blot.

Our NMR data, showing significantly reduced expression of pyruvate and lactate in ABCB5-KD G3361 melanoma cells, indicated a potential role of ABCB5 in regulating the glycolysis pathway as well as the Warburg effect, a metabolic hallmark of most cancer cells [24, 25]. In order to address the molecular mechanism underlying ABCB5-mediated regulation of these metabolic pathways, we blocked ABCB5 protein function in human G3361 melanoma cells through treatment with different concentrations of the anti-ABCB5 blocking monoclonal antibody (mAb) clone 3C2-1D12 (25 or 50 μg/ml), or, as controls, exposed cells to isotype control mAb or no treatment. Western blot analysis of lysates prepared from cells 48 h post-treatment demonstrated that mAb-mediated ABCB5 blockade resulted in markedly decreased expression of platelet isoform of phosphofructokinase (PFKP), the rate-limiting enzyme of glycolysis that catalyzes the irreversible conversion of fructose-6-phosphate to fructose-1,6-bisphosphate (Fig 4A). Quantitative analysis of these results using Image StudioTM Lite Software (LI-COR Biosciences) hereby revealed that compared to the corresponding isotype mAb treatment controls, and controlled for relative expression of beta-actin, the inhibition of ABCB5 in G3361 cells with 25 μg/ml anti-ABCB5 mAb resulted in a 32% decrease in PFKP expression, and with 50 μg/ml anti-ABCB5 mAb in a 84% decrease in PFKP expression, in a dose-dependent fashion. This finding, along with the result that expression of other enzymes upstream of PFKP in the glycolysis pathway remained unaffected by ABCB5 blockade, suggests that ABCB5 regulates the glycolysis pathway at the level of PFKP, thereby explaining the observed reduced levels of the glycolytic by-products pyruvate and lactate in ABCB5-KD melanoma cells. We have recently demonstrated regulation of an IL-1β/IL8/CXCR1 cytokine signaling circuit by ABCB5 [2]. Considering the compelling evidence regarding IL-1β-mediated regulation of HIF-1α [26], a key regulator of PFKP and the glycolysis pathway in cancer cells [27], we further dissected if the IL-1β-HIF-1α signaling cascade is involved in ABCB5-mediated regulation of glycolysis. ABCB5 mAb-treated G3361 cells showed significant attenuation of HIF-1α expression (Fig 4B). Together with our recent demonstration of ABCB5-mediated regulation of IL-1β secretion in human melanoma cells [2], these results suggest that the glycolysis pathway in melanoma cells is potentially regulated by an ABCB5/IL-1β/HIF-1α/PFKP signaling cascade. This finding is further supported by our results of induced expression of HIF-1α and PFKP in G3361 melanoma cells in the presence of exogenous IL-1β (Fig 5A and 5B). While the results of our pilot study provide initial evidence for the suggested mechanism, they also warrant further mechanistic studies aimed at elucidating additional details with the aim to establish a complete mechanistic picture.

### Behavior of energy metabolism

It is notable that no differences between ABCB5-WT and ABCB5-KD melanoma cells were detected for any of the phosphorylated high or low-energy metabolites quantified; this concerns PCr, nucleoside triphosphates (NTP; predominantly ATP), nucleoside diphosphates (NDP; predominantly ADP), and inorganic phosphate (P_i_). This invariance points to a remarkable stability of the cellular energetic state with respect to changing ABCB5 expression. Yet, identical energetic states for ABCB5-WT and ABCB5-KD cells were not to be expected in view of the significant changes observed for all three pathways involved in providing substrates for energy metabolism: (i) glycolysis (increased lactate and pyruvate levels), (ii) glutaminolysis (moderately increased glutamate, aspartate and alanine levels), and (iii) the tricarboxylic acid cycle (increased fumarate and succinate levels), as presented in the previous paragraph. One explanation for this discrepancy may be found in the nature of the ABCB5 protein. Since this molecule is an ATP-dependent efflux transporter, its expression may lead to increased energy-consuming transport activities across the plasma membrane. In this way, the increased energy provided by the three aforementioned pathways may be used for boosting transport processes, and the net effect may result in an unchanged energetic state. Note that for corresponding concentrations of PCr and PCho, similar values were obtained based on ^1^H and ^31^P NMR spectroscopy, as was to be expected (Tables A and B in S1 File, respectively). Minor differences can be ascribed to significant overlap with other resonances in ^1^H NMR spectra.

### Effects of ABCB5 expression on phospholipids

Although the overall amount of cellular PLs per total protein was not changed by ABCB5 expression in G3361 cells, some redistributions were observed within the PL pool (Fig 3 and Table 3; Tables C and D in S1 File). Notably, the relative amounts of sphingomyelin (SM), phosphatidylcholine (PtdCho), ethanolamine plasmalogen (PtdEtn_pl) and an unidentified PL ("b") were significantly lower in ABCB5-WT *vs*. ABCB5-KD melanoma cells, whereas the relative amounts of alkyl-acyl PtdCho (AAPtdCho) and another PL subgroup (PtdCho_u_tot_ = sum of PtdCho derivatives PtdCho_u_1_ to PtdCho_u_4_) were significantly higher. Furthermore, lower levels of cardiolipin (CL) in ABCB5-WT *vs*. ABCB5-KD cells was close to significance (p = 0.06). In general, these changes were moderate (by 10–20%). Based on its vicinity to the CL ^31^P NMR signal, the "b" signal most likely stems from a CL-derived PL subgroup; the apparently large statistical difference in "b" levels between ABCB5-WT and ABCB5-KD cells is due to the fact that this PL was detected in one ABCB5-WT sample only ("b" levels were set to zero for the other ABCB5-WT samples). When assessing the differences in PtdCho and PtdCho_u_tot_ levels one should take into consideration that the NMR signals representing these PL groups overlapped significantly, limiting the precision of signal separation. Therefore, values of the sums of PtdCho PLs (sum_PtdCho) are more robust than individual PtdCho and PtdCho_u_tot_ values. Note that AAPtdEtn and several low-abundance PL classes that remain unassigned for now ("a", "b", n_2_, n_3_, n_4_, u_1_, u_2_) were hardly detectable in ABCB5-KD cells, but were prevalent more consistently in ABCB5-WT cells. Thus, ABCB5 expression appears to stimulate the expression of certain PLs that tend to be at or below the detection threshold in a state of reduced ABCB5 expression. Since both ABC transporters and PLs are located in cellular membranes, the ABCB5-induced PLs may be functionally important for the ABCB5 transporter. More detailed interpretations will have to await the exact identification of the low-abundance PL classes in question.

It is interesting to note that products of PL degradation rather than PL synthesis were present at higher levels in ABCB5-expressing cells, and that this was accompanied by a higher abundance of glucose breakdown products. Overall, differences observed for both PLs and PL metabolites (see above) hint at alterations of membrane PL composition and turnover [28, 29] caused by ABCB5 expression. A list of all phospholipids and water-soluble metabolites quantified in this study, including their chemical shifts, is provided in form of an EXCEL table in S1 Table.

## Discussion

This comprehensive study presents, for the first time, the relevance of the expression of a cell surface marker of melanoma-initiating cells, ABCB5, for the cellular metabolome. Our results indicate overall higher levels of water-soluble metabolites due to expression of ABCB5 in normoxic G3361 melanoma cells, notably for several compounds involved in glycolysis and phospholipid metabolism, but also for some organic acids, such as amino acids. In addition, minor but significant redistribution within the phospholipid pool was observed. The statistical significance of metabolic differences, many of which were too moderate to be detected unambiguously by visual inspection of NMR spectra, was confirmed by appropriate tests. Our findings hint at specific biochemical processes that are altered as a consequence of ABCB5 expression.

The observed higher levels of glycolysis metabolites in ABCB5-expressing melanoma cells are in agreement with most earlier studies carried out on other cancer stem cells and stem-like cancer cells, as well as on cancer cells expressing ABC transporters other than ABCB5 [7–12]. Although all of these papers have reported enhanced glycolytic activity, there are only very few presentations of actual metabolomic results, and none of phospholipid profiles. Specifically, the data of the present report exhibit initial mechanistic evidence of glycolysis modulation by ABCB5 through an ABCB5/IL-1β/HIF-1α/PFKP signaling cascade.

Fermentative glycolysis is generally increased in cancer cells *vs*. normal cells, even if there is sufficient oxygen available for ATP production through oxidative phosphorylation (Warburg effect [30]). In hypoxic tumor niches where stem cells tend to reside, even more glycolysis is to be expected due to suppression of oxidative phosphorylation. We found that besides glycolytic metabolites, also the PDE levels were significantly increased by expression of the MIC marker ABCB5 for G3361 melanoma cells under normoxic culture conditions. PDEs are formed by PL degradation through phospholipases involved in membrane PL turnover. In agreement with a mechanism described elsewhere [28], enhanced PDE levels may indicate that PL homeostasis is 'primed' by ABCB5 as to facilitate membrane PL synthesis under conditions where CSCs thrive, e.g., under hypoxia. Indeed, hypoxia has been shown to increase the expression of choline kinase, a PL metabolism enzyme that is linked to cell survival and proliferation [5]. Thus, both glycolysis and PL metabolism appear to be primed for growth by ABCB5 expression in G3361 melanoma cells. This evidence is consistent with the interpretation that the metabolic profile of ABCB5-expressing cells and, potentially, multidrug-resistant cells and CSCs in general, is more tumor-like than that of cancer cells that are neither CSCs nor express MDR proteins. Future research should reveal if such a metabolic shift is generally common to ABC transporter-expressing side-population cells when compared to their corresponding non-expressing main-population cells as identified by flow cytometry.

We have previously demonstrated for several variants of the WEHI7.2 thymic lymphoma cell line that increases in antioxidant defense and drug resistance, conferred by a variety of transfection and selection techniques, resulted in more tumor-like metabolic profiles [31]. The resulting metabolic shifts to increased glycolytic and, potentially, glutaminolytic activity were very similar among the variants produced [32]. Thus, augmented glycolysis and glutaminolysis also characterize the metabolic phenotype of at least some cell lines that are steroid-resistant due to a bolstered antioxidant defense, although details of the underlying resistance mechanisms are currently unknown [33]. Consequently, increased glycolysis and glutaminolysis as characteristic traits of drug resistance do not appear to be limited to CSCs and/or cells with increased MDR protein expression. In fact, concurrent upregulation of ABC transporters, metabolic activity and antioxidant protection has recently been observed in the side population of a head and neck squameous cell carcinoma (HNSCC) mutant; here, cellular GSH levels were higher in cisplatin-resistant p53mut_c cells, consistent with a higher capacity to fend off cytotoxic oxidative effects such those caused by cisplatin treatment [34]. As opposed to glycolysis and glutaminolysis, energy metabolite levels were not significantly affected by ABCB5 expression in G3361 melanoma cells, whereas increasing steroid resistance in WEHI7.2 cells generally resulted in improved ATP production [31, 32].

Increased phospholipid turnover may contribute to steroid resistance in some WEHI7.2 variants, as was reported previously [35]. This is concurrent with our present finding that expression of the MIC marker ABCB5 modified PL metabolism of G3361 melanoma cells in a similar characteristic manner under normoxic conditions. PL metabolomics, like all "omics" methods, being a hypothesis-generating rather than a hypothesis-testing method (36), future studies will show whether this result is positively linked to facilitated PCho formation, membrane PL synthesis and cell proliferation under hypoxic conditions. If our hypothesis is confirmed, PL homeostasis should be considered as an additional putative target for overcoming CSC resistance to treatment, in addition to glycolysis and, potentially, glutaminolysis.

## Conclusions

Glycolysis, glutaminolysis and phospholipid metabolism were significantly modulated by the expression of the ATP-binding cassette transporter, ABCB5, a cell-surface marker for melanoma initiating cells. Combined with further biochemical results, our metabolomic study suggests initial evidence for an ABCB5-dependent IL1β—mediated signaling pathway for the regulation of glycolysis in human melanoma cells, thus opening new avenues for future research projects elucidating additional mechanistic details. While this metabolomic pilot study does not offer a complete mechanistic picture, it clearly warrants further investigations into the underlying signaling, metabolic, proteomic and, potentially, epigenetic processes. Overall, our results suggest that the biochemical pathways involved may offer targets for melanoma therapy, potentially in combination with other forms of treatment. Further studies will reveal if this approach can be extended to additional cancer cell lines expressing ABCB5, or to alternative, related ABC transporters.

## Supporting Information

S1 File Supplementary Information and Data. Figures A-C, Tables A-D.
(PDF)

S1 Table List of NMR chemical shifts used for metabolite analysis and quantification; abbreviations. NMR chemical shifts used for metabolite quantitation.
(XLSX)
