# Supplementary material for: Expression of Cell-Surface Marker ABCB5 Causes Characteristic Modifications of Glucose, Amino Acid and Phospholipid Metabolism in the G3361 Melanoma-Initiating Cell Line
Source: PLoS One. 2016 Aug 25;11(8):e0161803. doi: 10.1371/journal.pone.0161803 (PMC4999280; doi:10.1371/journal.pone.0161803)
Supplement: S1 File — Figures A-C, Tables A-D. (PDF) [file pone.0161803.s001.pdf]

Expression of Cell-Surface Marker ABCB5 Causes Characteristic Modifications of Glucose, Amino Acid and Phospholipid Metabolism in the G3361 Melanoma-Initiating Cell Line

Norbert W. Lutz, Pallavi Banerjee, Brian J. Wilson, Jie Ma, Patrick J. Cozzone, Markus H. Frank

S1 File Supplementary Information and Data

Figures A-C, Tables A-D

Use 'bookmarks' for quick overview of, and direct access to items contained in this document

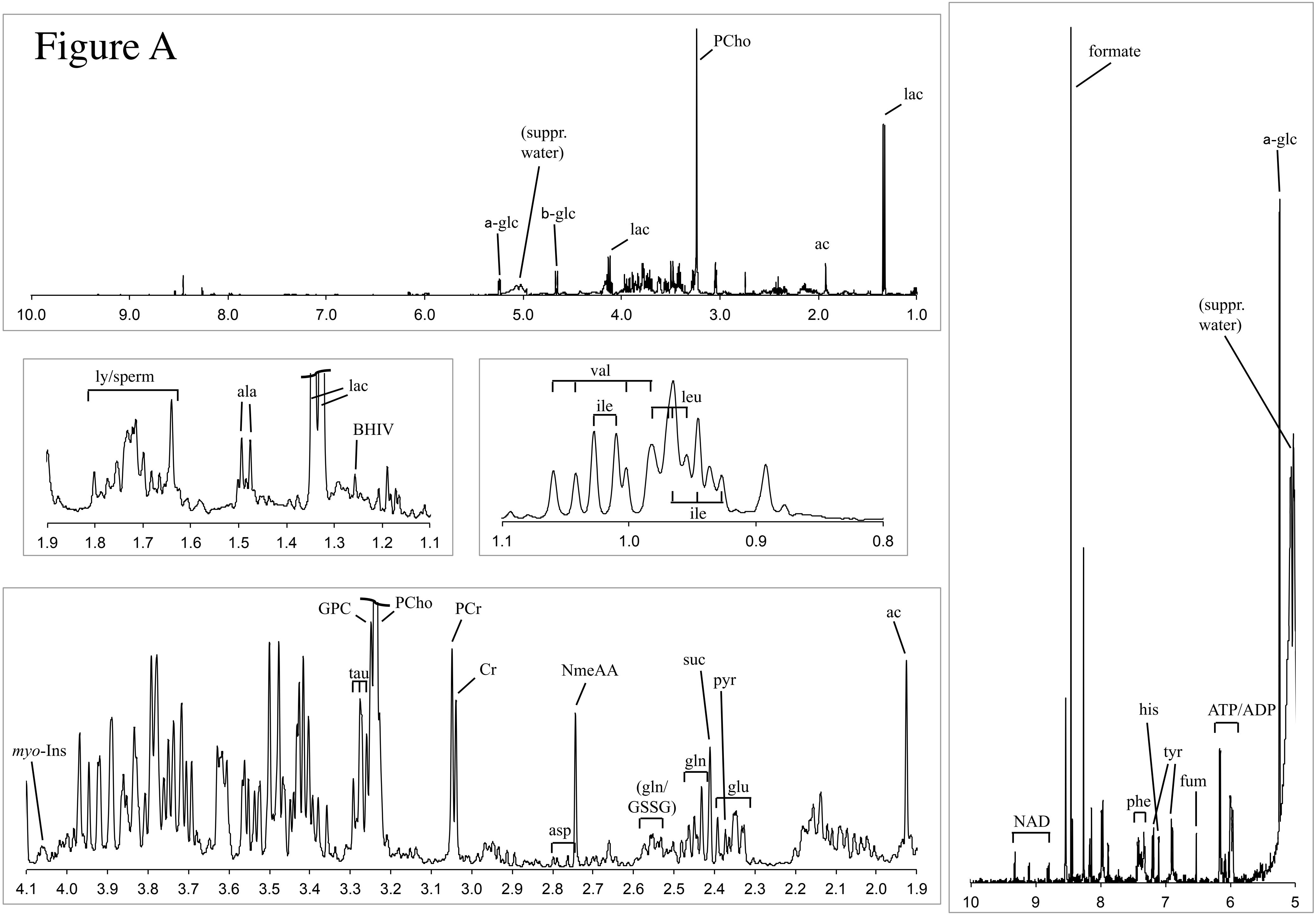

Figure A
PCho
lac
(suppr.
water)
a-glc
b-glc
lac
ac
10.0
9.0
8.0
7.0
6.0
5.0
4.0
3.0
2.0
1.0
ly/sperm
ala
lac
BHIV
1.9
1.8
1.7
1.6
1.5
1.4
1.3
1.2
1.1
val
ile
leu
ile
1.1
1.0
0.9
0.8
GPC
PCho
PCr
ac
tau
Cr
NmeAA
suc
pyr
myo-Ins
gln
(gln/
GSSG)
glu
asp
4.1
4.0
3.9
3.8
3.7
3.6
3.5
3.4
3.3
3.2
3.1
3.0
2.9
2.8
2.7
2.6
2.5
2.4
2.3
2.2
2.1
2.0
1.9
formate
a-glc
(suppr.
water)
his
ATP/ADP
tyr
fum
phe
NAD
10
9
8
7
6
5

Figure B

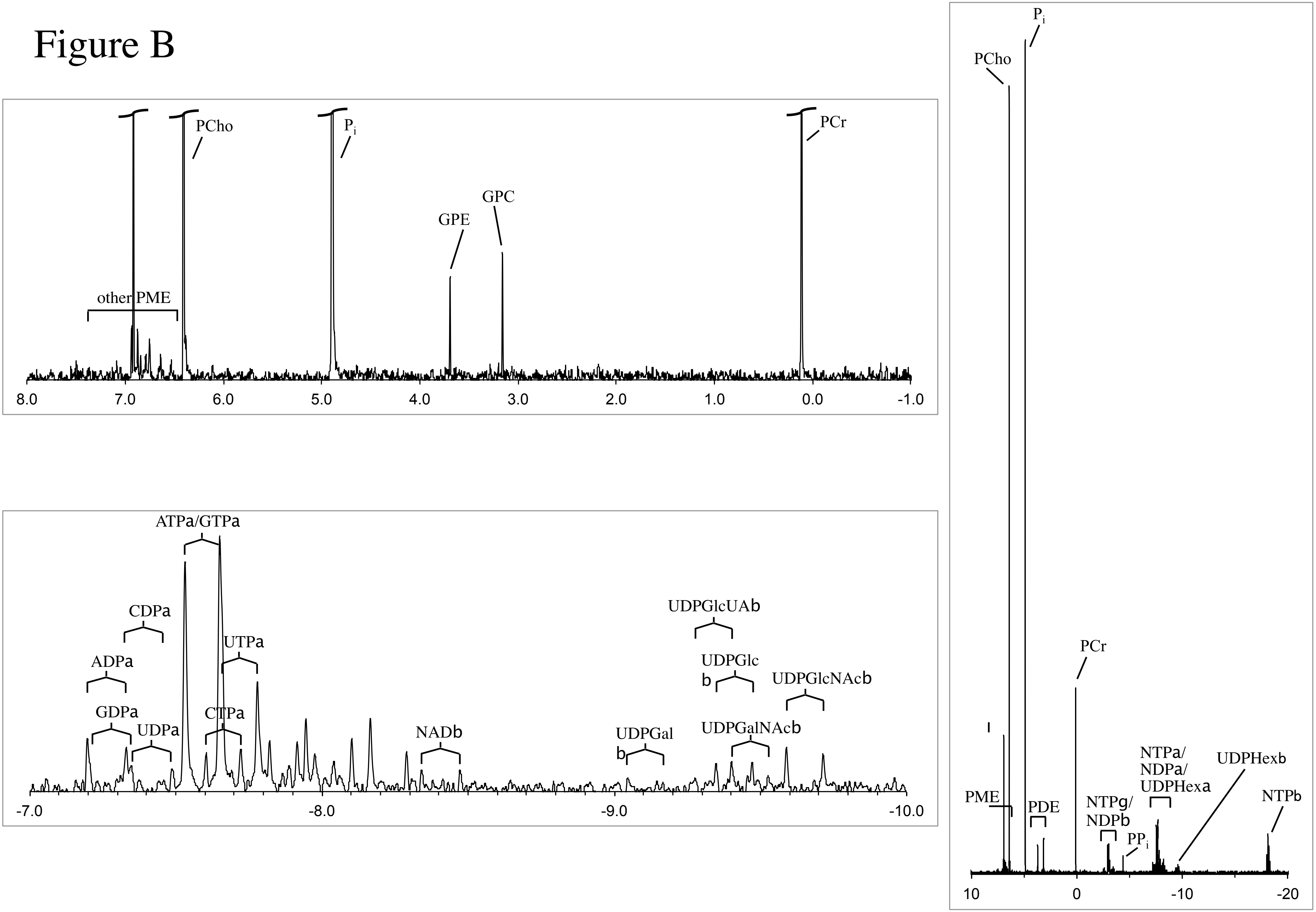
PCho
P_i
PCr
GPE
GPC
other PME
8.0
7.0
6.0
5.0
4.0
3.0
2.0
1.0
0.0
-1.0
ATPa/GTPa
CDPa
UTPa
ADPa
GDPa
UDPa
CTPa
NADb
UDPGal b
UDPGlcUAb
UDPGlc b
UDPGalNAcb
UDPGlcNAcb
-7.0
-8.0
-9.0
-10.0
PME
PDE
NTPg/ NDPb
PP_i
NTPa/ NDPa/ UDPHexa
UDPHexb
NTPb
10
0
-10
-20

Figure C

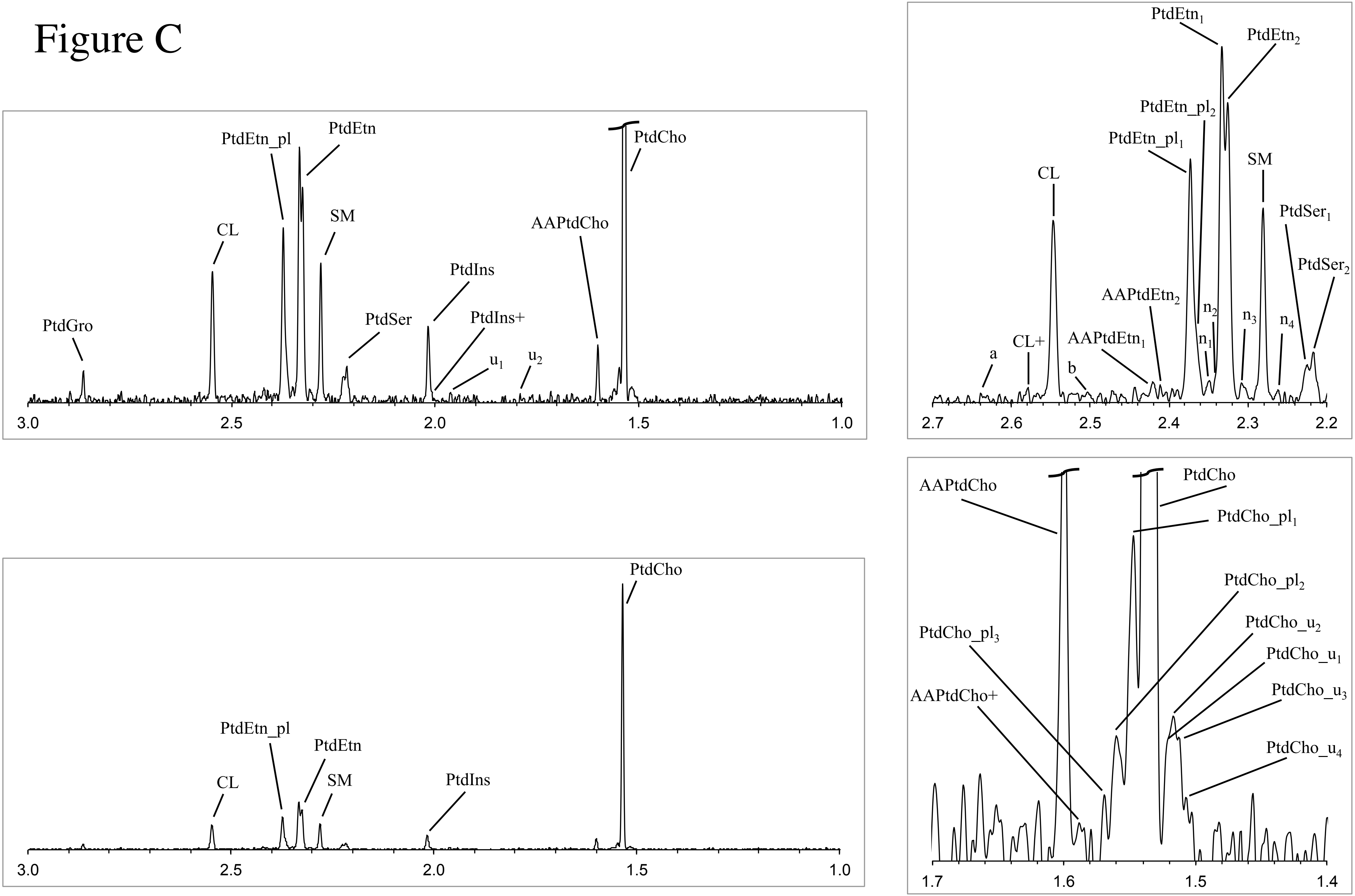
PtdGro
CL
PtdEtn_pl
PtdEtn
SM
PtdSer
PtdIns
PtdIns+
u$_1$
u$_2$
AAPtdCho
PtdCho
3.0
2.5
2.0
1.5
1.0
a
CL+
CL
b
AAPtdEtn$_1$
AAPtdEtn$_2$
PtdEtn_pl$_1$
PtdEtn_pl$_2$
PtdEtn$_1$
PtdEtn$_2$
n$_1$
n$_2$
n$_3$
n$_4$
SM
PtdSer$_1$
PtdSer$_2$
2.7
2.6
2.5
2.4
2.3
2.2
CL
PtdEtn_pl
PtdEtn
SM
PtdIns
PtdCho
3.0
2.5
2.0
1.5
1.0
AAPtdCho
PtdCho
PtdCho_pl$_1$
PtdCho_pl$_2$
PtdCho_pl$_3$
AAPtdCho+
PtdCho_u$_2$
PtdCho_u$_1$
PtdCho_u$_3$
PtdCho_u$_4$
1.7
1.6
1.5
1.4

Table A. Levels of further water-soluble metabolites in ABCB5-WT and ABCB5-KD G3361 melanoma cells.

Absolute concentrations, nmol/mg total protein

metabolite	ac	NmeAA	BHIV	gln	glc
ABCB5-WT	2.09 ± 0.16	1.96 ± 0.13	0.048 ± 0.006	5.55 ± 0.30	7.69 ± 0.67
ABCB5-KD	2.18 ± 0.43	1.73 ± 0.22	0.058 ± 0.006	5.29 ± 0.46	8.15 ± 0.87

metabolite	Cr	PCr	tau	myo-Ins	val
ABCB5-WT	3.36 ± 0.47	3.71 ± 0.69	10.82 ± 0.84	1.05 ± 0.12	0.59 ± 0.06
ABCB5-KD	2.75 ± 0.18	3.18 ± 0.20	9.79 ± 0.63	1.04 ± 0.11	0.58 ± 0.07

metabolite	ile	ly/sperm	leu	tyr
ABCB5-WT	1.07 ± 0.11	4.98 ± 0.66	0.43 ± 0.03	0.45 ± 0.05
ABCB5-KD	1.03 ± 0.12	5.02 ± 0.60	0.50 ± 0.05	0.47 ± 0.05

metabolite	his	phe
ABCB5-WT	0.34 ± 0.04	0.66 ± 0.08
ABCB5-KD	0.36 ± 0.04	0.65 ± 0.05

Relative concentrations, % of total metabolite protons

metabolite	lac*	ac	NmeAA	gln	suc
ABCB5-WT	12.60 ± 0.38	0.75 ± 0.05	0.71 ± 0.05	1.34 ± 0.10	0.34 ± 0.02
ABCB5-KD	11.48 ± 1.13	0.87 ± 0.16	0.69 ± 0.07	1.41 ± 0.10	0.29 ± 0.02

metabolite	glc	glu	Cr	PCr	PCho
ABCB5-WT	1.11 ± 0.17	1.67 ± 0.09	1.22 ± 0.19	1.34 ± 0.26	14.44 ± 0.96
ABCB5-KD	1.09 ± 0.11	1.59 ± 0.14	1.10 ± 0.07	1.28 ± 0.07	13.20 ± 0.90

metabolite	tau	myo-Ins	val	ile	ly/sperm
ABCB5-WT	2.62 ± 0.27	0.13 ± 0.01	0.21 ± 0.01	0.38 ± 0.03	1.18 ± 0.11
ABCB5-KD	2.61 ± 0.06	0.14 ± 0.01	0.23 ± 0.02	0.41 ± 0.03	1.32 ± 0.09

metabolite	fum	tyr	phe
ABCB5-WT	0.037 ± 0.004	0.108 ± 0.009	0.39 ± 0.04
ABCB5-KD	0.029 ± 0.002	0.124 ± 0.008	0.44 ± 0.03

Values determined by ^{1}H NMR spectroscopy, given as mean ± SEM; see also Table 1. For these compounds, statistical significance of differences between groups was absent. For abbreviations see S1 Table. *Includes minor contribution from threonine.

Table B. Levels of further phosphorylated water-soluble metabolites in ABCB5-WT and ABCB5-KD G3361 melanoma cells.

Absolute concentrations, nmol/mg total protein

metabolite	PCho	Pi	PCr	NDP
ABCB5-WT	14.82 ± 1.13	14.45 ± 2.39	4.06 ± 0.29	0.72 ± 0.06
ABCB5-KD	11.95 ± 0.90	14.60 ± 2.52	3.60 ± 0.26	0.71 ± 0.12

metabolite	UDP-hex	PPi	NTP
ABCB5-WT	1.26 ± 0.13	0.51 ± 0.09	5.26 ± 0.49
ABCB5-KD	1.03 ± 0.08	0.27 ± 0.08	4.50 ± 0.27

Relative concentrations, % of total metabolite phosphate

metabolite	PCho	Pi	PCr	NDP
ABCB5-WT	23.56 ± 0.45	22.89 ± 3.45	6.46 ± 0.19	1.14 ± 0.05
ABCB5-KD	21.26 ± 1.35	25.80 ± 3.84	6.41 ± 0.38	1.26 ± 0.20

metabolite	UDP-hex	NTP
ABCB5-WT	2.00 ± 0.16	8.36 ± 0.51
ABCB5-KD	1.82 ± 0.06	8.01 ± 0.38

Values determined by ^{31}P NMR spectroscopy, given as mean ± SEM; see also Table 2. NDP, NTP and UDP-hex levels were determined based on their α, β and β ^{31}P NMR resonances, respectively. For these compounds, statistical significance of differences between groups was absent. For abbreviations see S1 Table.

Table C. Further phospholipid levels in ABCB5-WT and ABCB5-KD G3361 melanoma cells.

PL class	PtdGro	a	CL	AAPtdEtn$_1$	AAPtdEtn$_2$
ABCB5-WT	0.26 ± 0.02	0.023 ± 0.014	0.68 ± 0.02	0.151 ± 0.074	*0.025*
ABCB5-KD	0.28 ± 0.03	*0.027*	0.72 ± 0.09	*0.013*	*0.030*

PL class	AAPtdEtn$_{tot}$	PtdEtn_pl$_1$	PtdEtn_pl$_2$	n$_1$	n$_2$
ABCB5-WT	0.18 ± 0.07	1.61 ± 0.05	0.59 ± 0.15	0.053 ± 0.015	0.007 ± 0.007
ABCB5-KD	*0.04*	1.78 ± 0.16	0.73 ± 0.13	0.039 ± 0.020	*0.043*

PL class	n$_3$	n$_4$	PtdEtn$_1$	PtdEtn$_2$	PtdEtn$_{tot}$
ABCB5-WT	0.067 ± 0.023	0.053 ± 0.021	2.06 ± 0.08	2.21 ± 0.15	4.26 ± 0.12
ABCB5-KD	*0.023*	n.d.	2.04 ± 0.26	2.23 ± 0.07	4.27 ± 0.24

PL class	PtdSer$_1$	PtdSer$_2$	PtdSer$_{tot}$	PtdIns	PtdIns+
ABCB5-WT	0.32 ± 0.02	0.45 ± 0.05	0.77 ± 0.06	0.66 ± 0.02	0.11 ± 0.06
ABCB5-KD	0.35 ± 0.11	0.42 ± 0.10	0.77 ± 0.07	0.70 ± 0.09	*0.01*

PL class	PtdIns$_{tot}$	u$_1$	u$_2$	AAPtdCho	AAPtdCho+
ABCB5-WT	0.78 ± 0.05	0.048 ± 0.028	*0.020*	0.42 ± 0.02	0.65 ± 0.02
ABCB5-KD	0.71 ± 0.09	n.d.	n.d.	0.35 ± 0.05	n.d.

PL class	PtdCho_pl$_1$	PtdCho_pl$_2$	PtdCho_pl$_3$	PtdCho_pl$_{tot}$	PtdCho_u$_1$
ABCB5-WT	0.50 ± 0.15	0.10 ± 0.06	*0.004*	0.61 ± 0.19	1.12 ± 0.14
ABCB5-KD	0.30 ± 0.06	0.05 ± 0.02	0.042 ± 0.021	0.39 ± 0.10	n.d.

PL class	PtdCho_u$_2$	PtdCho_u$_3$	PtdCho_u$_4$	sum_PtdCho	tot_PL
ABCB5-WT	0.27 ± 0.10	0.067 ± 0.049	0.22 ± 0.16	10.71 ± 0.38	22.51 ± 0.77
ABCB5-KD	0.36 ± 0.18	0.114 ± 0.094	0.11 ± 0.06	9.61 ± 0.82	22.78 ± 1.28

Values determined by ^{31}P NMR spectroscopy, given as mean ± SEM; see also Table 3. Absolute concentrations, nmol/mg total protein. Group sizes: n = 4 for ABCB5-WT; n = 3 for ABCB5-KD. Values in italics: PL class or subclass only detected in one extract of this group. n.d.: not detectable in any extract of this group. Table also includes PL subclasses. For these PLs, statistical significance of differences between groups was absent, or difficult to determine due to concentrations near the detection threshold. For abbreviations see S1 Table.

Table D. Further phospholipid levels in ABCB5-WT and ABCB5-KD G3361 melanoma cells.

PL class	PtdGro	a	CL	$AAPtdEtn_1$	$AAPtdEtn_2$
ABCB5-WT	1.17 ± 0.04	0.10 ± 0.06	6.04 ± 0.18	0.67 ± 0.34	*0.11*
ABCB5-KD	1.21 ± 0.07	*0.13*	6.31 ± 0.45	*0.06*	*0.15*

PL class	$AAPtdEtn_{tot}$	$PtdEtn\_pl_1$	$PtdEtn\_pl_2$	n_1	n_2
ABCB5-WT	0.78 ± 0.33	7.18 ± 0.41	2.59 ± 0.54	0.24 ± 0.07	0.06 ± 0.03
ABCB5-KD	*0.21*	7.80 ± 0.53	3.18 ± 0.50	0.20 ± 0.07	*0.21*

PL class	n_3	n_4	$PtdEtn_1$	$PtdEtn_2$	$PtdEtn_{tot}$
ABCB5-WT	0.29 ± 0.10	0.23 ± 0.09	9.18 ± 0.48	9.77 ± 0.40	18.95 ± 0.11
ABCB5-KD	*0.10*	n.d.	8.86 ± 0.65	9.86 ± 0.71	18.72 ± 0.28

PL class	$PtdSer_1$	$PtdSer_2$	$PtdSer_{tot}$	PtdIns	PtdIns+
ABCB5-WT	1.42 ± 0.11	2.00 ± 0.19	3.42 ± 0.28	2.96 ± 0.16	0.48 ± 0.26
ABCB5-KD	1.55 ± 0.49	1.83 ± 0.39	3.38 ± 0.17	3.04 ± 0.24	*0.07*

PL class	$PtdIns_{tot}$	u_1	u_2	AAPtdCho	AAPtdCho+
ABCB5-WT	3.44 ± 0.17	0.20 ± 0.12	*0.09*	1.87 ± 0.07	0.22 ± 0.07
ABCB5-KD	3.10 ± 0.22	n.d.	n.d.	1.55 ± 0.18	n.d.

PL class	$PtdCho\_pl_1$	$PtdCho\_pl_2$	$PtdCho\_pl_3$	$PtdCho\_pl_{tot}$	$PtdCho\_u_1$
ABCB5-WT	2.30 ± 0.73	0.47 ± 0.28	*0.02*	2.79 ± 0.91	4.95 ± 0.45
ABCB5-KD	1.32 ± 0.23	0.21 ± 0.11	0.19 ± 0.10	1.72 ± 0.43	n.d.

PL class	$PtdCho\_u_2$	$PtdCho\_u_3$	$PtdCho\_u_4$
ABCB5-WT	1.18 ± 0.42	0.31 ± 0.24	0.92 ± 0.63
ABCB5-KD	1.49 ± 0.75	0.55 ± 0.46	0.51 ± 0.29

Values determined by ^{31}P NMR spectroscopy, given as mean ± SEM; see also Table 3. Relative concentrations, % of total PL phosphorus. Group sizes: n = 4 for ABCB5-WT; n = 3 for ABCB5-KD. Values in italics: PL class or subclass only detected in one extract of this group. n.d.: not detectable in any extract of this group. Table also includes PL subclasses. For these PLs, statistical significance of differences between groups was absent, or difficult to determine due to concentrations near the detection threshold. For abbreviations see S1 Table.
